# Estimating the number of children exposed to parental psychiatric disorders through a national health survey

**DOI:** 10.1186/1753-2000-3-6

**Published:** 2009-02-19

**Authors:** Diego G Bassani, Cintia V Padoin, Diane Philipp, Scott Veldhuizen

**Affiliations:** 1 Centre for Global Health Research, St Michael's Hospital, Toronto, ON, Canada; 2 Dalla Lana School of Public Health, University of Toronto, Toronto, ON, Canada; 3 Child Health and Evaluative Sciences, Hospital for Sick Children, Toronto, ON, Canada; 4 The Hincks-Dellcrest Centre, Gail Appel Institute, Toronto, ON, Canada; 5 Department of Psychiatry, McMaster University, Hamilton, ON, Canada; 6 Department of Psychiatry, University of Toronto, Toronto, ON, Canada; 7 Centre for Addiction and Mental Health, Health Systems Research and Consulting Unit, Toronto, ON, Canada

## Abstract

**Objective:**

Children whose parents have psychiatric disorders experience an increased risk of developing psychiatric disorders, and have higher rates of developmental problems and mortality. Assessing the size of this population is important for planning of preventive strategies which target these children.

**Methods:**

National survey data (CCHS 1.2) was used to estimate the number of children exposed to parental psychiatric disorders. Disorders were diagnosed using the World Psychiatric Health Composite International Diagnostic Interview (WMH-CIDI) (12 month prevalence). Data on the number of children below 12 years of age in the home, and the relationship of the respondents with the children, was used to estimate exposure. Parent-child relations were identified, as was single parenthood. Using a design-based analysis, the number of children exposed to parental psychiatric disorders was calculated.

**Results:**

Almost 570,000 children under 12 live in households where the survey respondent met criteria for one or more mood, anxiety or substance use disorders in the previous 12 months, corresponding to 12.1% of Canadian children under the age of 12. Almost 3/4 of these children have parents that report receiving no mental health care in the 12 months preceding the survey. For 17% of all Canadian children under age 12, the individual experiencing a psychiatric disorder is the only parent in the household.

**Conclusion:**

The high number of children exposed causes major concern and has important implications. Although these children will not necessarily experience adversities, they possess an elevated risk of accidents, mortality, and of developing psychiatric disorders. We expect these estimates will promote further research and stimulate discussion at both health policy and planning tables.

## Introduction

Children of parents with mental illness (MI) are shown to have higher mortality rates, as well as an increased risk of developing a wide range of mental and addictive disorders [1]. Prevention programs and policies that have been developed to target these children are effective [2-11] and it is known that many of these youngsters can do reasonably well with appropriate support, but without a clear understanding of the size of this population and its demographics, efforts aimed at improving their situation or limiting their exposure are seriously restricted.

There is a significant body of literature demonstrating that exposure to parental psychopathology puts children at risk of untoward outcomes. For example, children of parents with either depression [12-14], schizophrenia [15], or substance abuse or dependence [16,17], are at higher risk of developing the same respective condition as the parent. Non-substance related psychopathologies are also more common among children of substance abusers [18]. Similarly, children of parents with anxiety – [19], substance use – [16,20], and eating-disorders [21-23] are also at higher risk for psychopathology. Parental depression is also associated with impaired development [24], behaviour [25], physical health [26] and higher health service use [27,28]. Injuries are also more frequent among children of mothers with mental health problems [29].

Many factors may explain the risk, including genetic inheritance [30], parenting quality, patterns of stimulation, relationship factors [31,32] and other adverse experiences [33]. Compounding the situation further are single parent families where the guardian suffers from a MI or substance abuse, or families where both parents have a history of psychiatric disorders [14,34]. In these scenarios, the children are at an even greater risk of MI, substance abuse, death due to suicide, or drug overdose, as compared to children from two-parent families where only one parent experiences MI [1].

Despite significant documentation of these detrimental associations, and interventions aimed at their prevention, few studies have focused on quantifying the children at risk. In a US community-based sample of first-admission patients with diagnoses of Schizophrenia/Schizoaffective Disorder, Bipolar Disorder with psychotic features, and Major Depressive Disorder with psychosis, it was estimated that almost one third of first-admission psychiatric patients were parents [35]. In Australia, between 29% and 35% of female mental health service users are parents of children under 18 [36,37], and 70% of children living with MI parents were under 6 years of age [38], suggesting that a large proportion of patients receiving mental health services are in fact parents.

It should be noted, that of the few studies looking at the population of at-risk children, the vast majority have focused only on data from parents in treatment settings. Grant [39] completed one of the few studies that used population-based data; using data from the National Longitudinal Alcohol Epidemiological Survey (1992), it was estimated that approximately 1 in 4 American children under 17 are exposed to alcohol abuse or dependence in the family (lifetime). Past year exposure to parental alcohol abuse or dependence for children under 12 is about 10%. This study only measured exposure to alcohol abuse, and yet, identified a sizeable number of children affected. Unfortunately, no other studies using population samples have looked at exposure to parental MI.

The implementation of child mental health prevention programs requires that policy makers and practitioners become attentive to the large divergence between what is known and what is currently practised. It has been suggested [40] that in order to strengthen the link between research and practice in children's mental health, clearer strategic planning around prevention needs to be developed. As strategic planning requires characterization of the target population, adult patients under psychiatric intake should at the least be asked whether they have children. Furthermore, it should be noted that even if this strategy were adopted, children of parents who do not seek treatment – and who therefore may be at greater risk – would fall through the cracks.

Since estimates of the number of children exposed to parental MI in Canada are not available, the aim of this paper is to use data from the Canadian Community Health Survey cycle 1.2 – Mental Health and Well-Being (CCHS 1.2) to estimate the size of this population. As these children are at an increased risk of psychiatric disorders [41], defining the size of this population will hopefully serve as a basis for the planning of preventive mental health strategies targeting children.

## Methods

The CCHS 1.2 [42] was conducted in 2002 and collected information on the mental health and well-being of non-institutionalized individuals aged 15 years and older living in private occupied dwellings across the Canadian provinces, excluding those living on Crown lands and military bases. The survey was the first attempt to generate national estimates of the burden of mental illnesses in Canada. Using a two-stage stratified cluster design, the sample (n = 36,984) was allocated among provinces proportionally to the population in each province and is weighted to correspond to the general population of Canada (weighted n = approximately 24 million). One person was selected from each household and 98% of the targeted population was surveyed. The probability of selection for each person was defined as a function of the household composition. A detailed description of the survey methodology is available elsewhere [42]. The mean age of the sample was 43.7 years (s.d. 17.8), 50.7% of the respondents were women and 47.2% had a college degree while 25.4% had less than high-school education [43].

Psychiatric disorders were diagnosed according to a modified version of the World Mental Health Composite International Diagnostic Interview (WMH-CIDI) [42], using algorithms based on the 12 months preceding the interview. The CCHS 1.2 includes algorithms for the diagnosis of five mood and anxiety disorders: major depressive disorder, manic episode, panic disorder, social phobia and agoraphobia [44]. Information about frequency, quantity, related problems, and dependence symptoms of alcohol and illicit drug use was also collected, as were symptoms of substance dependence. Respondents were classified as having substance dependence if they reported three or more symptoms of substance dependence were reported during the previous 12 months [45].

Although the CCHS 1.2 covers the most prevalent psychiatric disorders, the criteria for the diagnosis of substance abuse, psychosis and personality disorders outlined in the 4^th ^edition of the Diagnostic and Statistical Manual of Mental Disorders' (DSM-IV) were not included in the survey by Statistics Canada. This decision was unfortunate, beyond our control, and may lead to an underestimation of the burden of mental disorders in Canada's adult population [43].

The information about household structure, including number of children and their relationship to the respondent, was collected in detail from one of the household members, but not necessarily from the individual interviewed. Using the Parent-child relationships could be identified and separated from other types of relationships in our analysis, as was the information about the presence of single parenthood.

The percent of respondents reporting symptoms of anxiety disorders, mood disorders, substance problems or dependence issues, and living in a household with children below the age of 12 years, was estimated using the survey data. The proportion of Canadian children whose parents experience each of the above categories was calculated using the survey parameters. Information about the composition of the family – where a parental relationship was present – was also used to calculate estimates by type of family (e.g. single-parent families) and also to exclude non-parental relationships (i.e. other adult individuals in the household that are not the child's parents). The proportion of children exposed to both treated and untreated parental mental disorder was also estimated.

All estimates were weighted to account for the design of the survey and several of the included complexities of over-representation, data imputation, and sampling probabilities [42]. The 95% confidence intervals are presented, and prevalence of exposure identified in the survey is projected to actual population numbers using the 2006 Canadian Census data. Analysis was conducted in Stata 9.0 SE; variances were estimated using the bootstrap weights provided by Statistics Canada. Informed consent was obtained by Statistics Canada previous to survey administration.

## Results

According to the 2006 Canadian Census, Canada has 4.5 million children under age 12. One in every ten children live with a parent that has a psychiatric disorder, and one in every six resides in a household where at least one individual has a psychiatric disorder (not necessarily the parent – data not shown). This corresponds to almost 570,000 Canadian children under age 12 experiencing parental psychiatric disorders (12.1% of all children under 12). Over 3/4 of these children (78.5%, or 446,405 children under age 12) have parents that report receiving no mental health care in the 12 months preceding the survey. For 17% of these children the individual experiencing a psychiatric disorder is the only parent in the household.

The majority of the children under 12 who are living with parents with psychiatric disorders are exposed to substance use disorders (10.0%:95%CI 9.3; 10.5). This diagnosis was the most common exposure both for children under 5 (9.8%:95%CI 8.4; 11.3) and from 5 to 11 (10.0%:95%CI 9.1; 11.6). Mood disorders and anxiety disorders were the diagnosis observed in the parents of 5.1% of all children under 12 and the prevalence was similar both for children under 5 as well as those between 5 and 11 years (Additional file 1, Table S1).

Most parents with substance use disorders reported not receiving treatment in the previous 12 months. Children of such parents comprise the majority of all exposed children (8.2%:95%CI 7.7; 9.3). The prevalence of exposure to untreated parental substance use disorders was 9.0% (95%CI 8.5; 9.7) for children under 5, and 8.0% (95%CI 7.4; 8.9) for children between 5 and 11 (Additional file 1, Table S2).

Over 33,000 Canadian children under 5 live in single-parent families and the parent has a psychiatric disorder corresponding to 1.5% (95%CI 0.8; 2.3) of all Canadian children under 5. The proportion increases for children between 5 and 11 (2.1%:95%CI 1.0; 2.9), with the overall prevalence reaching 2.0% (95%CI 1.1; 2.7), corresponding to almost 94,000 Canadian children under age 12 (Additional file 1, Table 3).

## Discussion

Certainly the most striking finding was that one in every ten Canadian children under 12 is living with a parent who has some form of psychiatric disorder. Furthermore, the vast majority of these parents report no mental health care in the previous 12 months. In addition, 1 in 6 children exposed children come from single parent homes – two factors which are cause for significant concern. While parental psychiatric disorders convey a risk to children in and of itself, it may also serve as an identifier for a series of adversities that also increase risk to offspring such as exposure to trauma, high-risk neighbourhoods, downward social mobility and poor social and economic support.

Whereas there has been no previous work of this scope to date, the estimate of exposure to past year alcohol abuse and dependence in American households, according to the 1992 National Longitudinal Alcohol Epidemiological Survey, is 10.25% for children under 12. Of these children, 70.4% were directly exposed to parental alcohol abuse or dependence, yielding a prevalence of exposure of 7.2% [39]. Our numbers for Canada indicate that the prevalence of exposure to parental substance use disorders, and alcohol abuse and dependence (i.e. excluding illicit substances) for children under 12 is 11.4% and 8.3%, respectively (data not shown).

Substance use disorders were the most common psychiatric disorder experienced by parents of children under 12. Research indicates that these children are at a higher risk of developing substance use disorders themselves, as well as non-substance related psychopathologies [18]. This may be due to the fact that parents who abuse alcohol are more likely to expose their children to a number of adverse events. Specifically, these children are at an increased risk of encountering emotional, sexual, and physical abuse, domestic violence, parental separation, incarceration, illicit drug use, witnessing suicide attempts, as well as a combination of more than one of the above adverse experiences [46]. These have been shown as strong predictors of future alcohol abuse and depression in children [46-50].

Similarly, population-based data shows that children (interviewed when adults) of parents with psychiatric symptoms appear to be at higher risk of not only the same disorder that the parent experienced, but also of most other disorders [34]. Furthermore, a recent longitudinal study has shown that children of parents with MI are at higher risk of mortality, which remains elevated from birth to early adulthood [14]. It has been suggested that the transmission – given the absence of better wording – of psychiatric disorders from parents to children can be categorized in two broad classes: anxiety and depression – or chronic dysphoric disorders – and 'acting-out' disorders, represented mainly by harmful substance use [51]. Although initial findings suggested that children of parents with disorders from one of these groups were only at higher risk of developing a disorder from the same group [19,51], this hypothesis has been questioned [52,53]. Thus, future strategies should perhaps be less focused on prevention or identification of risk factors for any specific diagnosis, but on broader arenas that may likely encompass improvement of parenting skills, child protection and follow-up. Furthermore, the finding that most children living with a parent affected by MI are also in single parent families, indicates the need for supportive strategies for these parents and children. Children from such families are at higher risk of MI, substance abuse, death due to suicide, and drug overdose [1], as compared to two-parent families with one mentally ill parent. Additionally, children from families where both parents have a history of psychiatric disorders, compared to cases where only one parent experienced psychopathology [14,34], are also at higher risk, indicating that there may be some shielding effect exerted by the parent without MI. This is confirmed by empirical observations of families where the presence of a father with no history of MI may buffer the effects of maternal psychopathology, and lower the children's risk of developing possible MI [54].

### Forecasting for a better future

Although the mechanisms through which parental MI influences children's mental health and development are not clearly understood, the presence of the association is well documented. However, the use of such evidence to generate policy and planning strategies aimed at reducing the burden carried by these children has been limited. Also, as it is estimated that only half of the burden of mental disorders can be reduced through currently available treatment modalities [55], the development of new preventive strategies has been suggested as a possible alternative [55,56]. Certainly, our findings suggest a significant population of children for whom such prevention programs should be targeted at in the hopes of reducing future burden.

### Study limitations

Most methodological limitations of the study indicate we may be underestimating the number of children exposed to parental psychiatric disorders. The CCHS 1.2 collected information on one adult respondent per household and may miss individuals that were homeless, hospitalized or living in institutions at the time of the survey. These individuals are more likely to have psychiatric disorders and if they were missed by the survey but do live in the household, we may be underestimating the proportion of children exposed to parental psychiatric disorders.

The fact that the survey did not collect information on the mental health status of other family members may also result in an underestimate of the number of children exposed to parental psychiatric disorders. The confidence intervals around the estimates were calculated using the weights that take into account non-response, probability of selection and the complex sampling scheme adopted by CCHS 1.2.

As mentioned earlier, the survey covers the most prevalent psychiatric disorders, but no all of them. For example, the survey did not include certain diagnosis such as psychosis and personality disorders. In addition, the criteria for the diagnosis of substance abuse outlined in the 4^th ^edition of the Diagnostic and Statistical Manual of Mental Disorders' (DSM-IV) were also not included in the survey by Statistics Canada. These decisions were beyond our control but may arguably lead to an underestimation of the burden of mental disorders in Canada's adult population [43] and as a consequence, to an underestimate of the number of children exposed to parental psychiatric disorders.

### The need for further studies

Family-oriented interventions to prevent adverse outcomes among children of parents experiencing MI are rare. However, it is encouraging to see that there is a growing body of literature evaluating the effectiveness of such strategies – geared towards various age groups – in reducing the incidence of MI [56-58]. Prevention programs such as the Incredible Years Program, a behavioural training program targeting parents in high risk families, have been well studied [6,7,59-64] and are known to improve parenting skills and parental interaction with the child. Interventions such as the Nurse-Family Partnership, an evidence-based, nurse home-visiting program for low-income, first-time parents and their children, have been able to reduce exposure of high-risk children to adverse events, and to prevent a series of developmental problems among these children, as well as in the overall target population [3-5,65-71].

Identifying these children has important implications. Child psychiatric disorders usually persist into adulthood [72,73], and prevention represents an opportunity to reduce health expenditures and promote sustainability of the health care system [74]. Identifying which children are at risk is also one of the possible keys to the success of the Nurse-Family Partnership, and its possible cost-effectiveness, once it has targeted these children. Other preventive strategies could be implemented by psychiatrists and other mental health professionals if the identification of the patients that are parenting small children was part of the routine of mental health service providers.

Finally, none of the adverse exposures are chosen by the children themselves, and neither are family composition or background. Coupled with the fact that these factors play an important role in child development and have far reaching effects into adulthood [1,75,76], this issue should raise awareness and promote action in child advocacy at the level of health professionals, as well as policy. Improving support for children and families with parental MI may be the key to enhancing protective factors and reducing risk of future morbidity. We hope that documenting the significant number of children exposed to parental psychiatric disorders serves as a stimulus for action that will foster safer and healthier development for them.

## Competing interests

The authors declare that they have no competing interests.

## Authors' contributions

DGB and SV Planned the study, conducted the statistical analysis and prepared the manuscript. DGB, CVP and DP Planned the study, reviewed the literature, discussed the results and analysis and prepared the manuscript. All authors read and approved the final manuscript.

## Supplementary Material

Additional file 1 **Tables.** This file contains Tables S1, S2 and S3. Click here for file
