# Supplementary material for: Estimating the number of children exposed to parental psychiatric disorders through a national health survey
Source: Child Adolesc Psychiatry Ment Health. 2009 Feb 19;3:6. doi: 10.1186/1753-2000-3-6 (PMC2647902; doi:10.1186/1753-2000-3-6)
Supplement: Additional file 1 — Tables. This file contains Tables S1, S2 and S3. [file 1753-2000-3-6-S1.pdf]

Table S1 - Proportion of Canadian children under 12 who have a parent with mental disorders.

	Proportion and number of children under 12 living with parents with selected MI					
**DSM-IV Diagnosis (12 months)**	**0-4**		**5-11**		**Total (under 12)**	
	**% (95%CI)**	**n[a]**	**% (95%CI)**	**n[a]**	**% (95%CI)**	**n[a]**
**Any Anxiety disorder**	**4.0 (3.1;4.8)**	**88,490**	**5.1 (3.9;5.7)**	**126,824**	**4.6 (3.8;5.3)**	**216,153**
Agoraphobia	0.6 (0.3;1.0)	13,616	0.7 (0.4;1.2)	17,186	0.7 (0.3;1.2)	30,803
Panic disorder	1.7 (1.2;1.9)	37,975	1.8 (1.1;2.0)	44,215	1.7 (1.2;2.5)	82,191
Social phobia	2.2 (1.9;2.8)	49,211	2.9 (2.3;3.5)	73,075	2.6 (2.0;3.1)	122,302
**Any Mood disorder**	**5.1 (4.7;5.6)**	**112,825**	**5.2 (4.6;5.9)**	**129,311**	**5.1 (4.8;5.7)**	**239,649**
Major Depression	4.3 (4.0;4.5)	95,348	4.5 (4.0;5.1)	110,739	4.4 (3.9;5.0)	206,091
Mania	1.0 (0.7;1.6)	22,130	0.9 (0.5;1.4)	22,373	0.9 (0.5;1.3)	44,501
**Any Substance use disorder***	**9.8 (8.4;11.3)**	**216,801**	**10.0 (9.1;11.6)**	**248,674**	**10.0 (9.3; 10.5)**	**469,900**
Alcohol Dependence	2.2 (1.8;2.7)	209,237	2.0 (1.5;2.7)	212,439	2.1 (1.8;2.7)	421,655
Substance Abuse	9.5 (8.9;9.9)	47,608	8.5 (7.8;9.0)	49,134	9.0 (8.5;9.6)	96,738
Illicit Drug Dependence	0.3 (0.1;0.7)	7,273	0.4 (0.2;0.7)	9,034	0.3 (0.1;0.7)	16,308
**TOTAL (Any Disorder)**	**12.3 (11.2;13.1)**	**272,107**	**11.9 (11.3;12.5)**	**295,922**	**12.1 (11.5;12.3)**	**568,578**

Note: Since multiple disorders are possible in the same individual, the same child may be exposed to more than one.
[a] total number of children estimated based on 2006 Census estimates.
*Problems or dependence

Table S2 - Proportion of Canadian children under 12 who have parents with mental disorders that did not receive mental health care in the previous 12 months.

DSM-IV Diagnosis (12 months)	***Proportion and number of children under 12 living with parents with selected untreated MI***					
	**0-4**		**5-11**		**Total (under 12)**	
	**% (95%CI)**	**n[a]**	**% (95%CI)**	**n[a]**	**% (95%CI)**	**n[a]**
**Any Anxiety disorder**	**2.3 (2.0;3.7)**	**50,882**	**1.9 (1.5;2.6)**	**47,248**	**2.2 (1.9;3.1)**	**103,378**
Agoraphobia 0.5	(0.2;0.7)	10,710	0.3 (0.1;0.8)	7,252	0.4 (0.1;0.7)	17,957
Panic disorder	1.1 (0.8;1.6)	23,936	0.5 (0.2;1.0)	13,666	0.8 (0.3;1.2)	37,590
Social phobia	1.4 (1.2;1.9)	30,295	1.5 (1.3;2.0)	38,245	1.5 (1.3;1.9)	68,544
**Any Mood disorder**	**1.4 (1.0;1.9)**	**30,972**	**1.7 (1.3;2.2)**	**42,275**	**1.5 (1.0;1.8)**	**70,485**
Major Depression	1.3 (0.7;1.7)	29,420	1.6 (1.1;2.0)	40,036	1.5 (1.0;1.9)	69,462
Mania 0.1	(0.0;0.4)	2,212	0.08(0.0;0.2)	1,989	0.1 (0.0;0.3)	4,699
**Any Substance use disorder***	**9.0 (8.5;9.7)**	**199,103**	**8.0 (7.4;8.9)**	**198,939**	**8.2 (7.7;9.3)**	**385,318**
Alcohol Dependence	1.8 (1.1;2.3)	178,844	1.4 (1.0;2.1)	181,583	1.6 (1.3;2.0)	360,409
Substance Abuse	8.1 (7.8;8.5)	38,877	7.2 (6.7;7.9)	35,051	7.7 (7.1;8.5)	73,919
Illicit Drug Dependence	0.1 (0.0;0.3)	2,828	0.1 (0.0;0.4)	2,487	0.1 (0.0;0.3)	4,699
**TOTAL (Any Disorder)**	**9.6 (8.9;10.2)**	**212,376**	**8.9(7.6;9.2)**	**221,320**	**9.5(9.0;10.1)**	**446,405**

Note: Since multiple disorders are possible in the same individual, the same child may be exposed to more than one.
[a] total number of children estimated based on 2006 Census of Canada estimates.
*Problems or dependence

Table S3 – Proportion of Canadian children under 12 who have single parents with mental disorders.

	Proportion and number of children under 12 living with single parents with selected MI					
**DSM-IV Diagnosis (12 months)**	**0-4**		**5-11**		**Total (under 12)**	
	**% (95%CI)**	**n[a]**	**% (95%CI)**	**n[a]**	**% (95%CI)**	**n[a]**
**Any Anxiety disorder**	**0.6 (0.1;1.6)**	**13,274**	**0.7 (0.3;1.5)**	**17,407**	**0.7 (0.3;1.1)**	**34,303**
Agoraphobia 0.1	(0.0;1.2)	1,107	0.1 (0.0; 1.7)	1,492	0.1 (0.0;0.8)	4,699
Panic disorder	0.3 (0.1;0.9)	6,999	0.6 (0.1;1.1)	14,907	0.5 (0.1; 1.0)	21,911
Social phobia	0.4 (0.1;1.1)	9,141	0.7 (0.2;1.3)	16,666	0.6 (0.1; 1.1)	25,812
**Any Mood disorder**	**1.1 (0.5;1.9)**	**24,335**	**1.3 (0.8;1.7)**	**32,328**	**1.2 (0.6; 1.9)**	**56,388**
Major Depression	1.0 (0.4;2.0)	20,956	1.3 (0.8; 1.6)	31,141	1.1 (0.5; 1.8)	52,104
Mania 0.3	(0.1;1.1)	5,613	0.2 (0.0; 1.1)	5,661	0.2 (0.0; 1.0)	11,273
**Any Substance use disorder***	**0.8 (0.2;1.4)**	**17,698**	**1.2 (0.7; 1.8)**	**29,841**	**1.1 (0.8; 1.5)**	**51,689**
Alcohol Dependence	0.7 (0.1;1.5)	14,402	0.9 (0.4; 1.3)	22,106	0.8 (0.5; 1.2)	36,514
Substance Abuse	0.2 (0.0;1.2)	4,565	0.4 (0.1; 1.0)	8,625	0.3 (0.0; 0.9)	13,193
Illicit Drug Dependence	0.1 (0.0;1.8)	1,106	0.1 (0.0; 1.6)	1,989	0.1 (0.0; 1.5)	2,819
**TOTAL (Any Disorder)**	**1.5 (0.8;2.3)**	**33,184**	**2.1 (1.0;2.9)**	**52,222**	**2.0 (1.3; 2.6)**	**93,980**

Note: Since multiple disorders are possible in the same individual, the same child may be exposed to more than one.
[a] total number of children estimated based on 2006 Census of Canada estimates.
*Problems or dependence
